# Computational quantification of global effects induced by mutations and drugs in signaling networks of colorectal cancer cells

**DOI:** 10.1038/s41598-021-99073-7

**Published:** 2021-10-01

**Authors:** Sara Sommariva, Giacomo Caviglia, Silvia Ravera, Francesco Frassoni, Federico Benvenuto, Lorenzo Tortolina, Nicoletta Castagnino, Silvio Parodi, Michele Piana

**Affiliations:** 1 Dipartimento di Matematica, Università di Genova, via Dodecaneso 35, 16146 Genoa, Italy; 2 Dipartimento di Medicina Sperimentale, Università di Genova, Via De Toni 14, 16132 Genoa, Italy; 3 Dipartimento di Medicina Interna, Università di Genova, via Leon Battista Alberti 2, 16132 Genoa, Italy

**Keywords:** Cellular signalling networks, Computational models, Biochemical networks, Numerical simulations, Cancer models

## Abstract

Colorectal cancer (CRC) is one of the most deadly and commonly diagnosed tumors worldwide. Several genes are involved in its development and progression. The most frequent mutations concern APC, KRAS, SMAD4, and TP53 genes, suggesting that CRC relies on the concomitant alteration of the related pathways. However, with classic molecular approaches, it is not easy to simultaneously analyze the interconnections between these pathways. To overcome this limitation, recently these pathways have been included in a huge chemical reaction network (CRN) describing how information sensed from the environment by growth factors is processed by healthy colorectal cells. Starting from this CRN, we propose a computational model which simulates the effects induced by single or multiple concurrent mutations on the global signaling network. The model has been tested in three scenarios. First, we have quantified the changes induced on the concentration of the proteins of the network by a mutation in APC, KRAS, SMAD4, or TP53. Second, we have computed the changes in the concentration of p53 induced by up to two concurrent mutations affecting proteins upstreams in the network. Third, we have considered a mutated cell affected by a gain of function of KRAS, and we have simulated the action of Dabrafenib, showing that the proposed model can be used to determine the most effective amount of drug to be delivered to the cell. In general, the proposed approach displays several advantages, in that it allows to quantify the alteration in the concentration of the proteins resulting from a single or multiple given mutations. Moreover, simulations of the global signaling network of CRC may be used to identify new therapeutic targets, or to disclose unexpected interactions between the involved pathways.

## Introduction

Colorectal cancer (CRC) is the second most common cancer in women (865,630 estimated incident cases worldwide, according to the GLOBALCAN 2020 data^1^, https://gco.iarc.fr/) and the third most common in men (1,065,960 estimated incident cases worldwide)^2,3^, representing one of the most significant causes of cancer death^4^. Both genomic and epigenetic alterations are common in CRC and are the driving forces of tumorigenesis.

CRC arises from one or more of three mechanisms: the chromosomal instability pathway, the microsatellite instability pathway, and the CpG island methylator phenotype. Focusing the attention on the chromosomal instability pathway, since 1990, it is well known that the mutations of genes involved in cell growth and differentiation pathways play a pivotal role in the development and progression of CRC^5^. Although numerous mutations have been associated with the CRC development and progression, the most frequent driver and gate-keeper mutations commonly found concern TP53, APC, KRAS, PTEN, SMAD4, PIK3CA, BRAF, AKT^2,3,6–9^. In addition, several indications suggest that the aggressiveness and malignancy of CRC depend on the mutation order. In particular, it has recently been observed that the most frequent mutation order in CRC is the following: APC, KRAS, SMAD4, and TP53^10^.

Studying only the chemical reactions directly involving the mutated proteins is not sufficient to provide biological insights into the changes induced in cell behavior by mutations^11–13^. Proteins are usually grouped into functional pathways that are designed to represent a defined cellular process^14^ and are often visualized through linear diagrams^15–17^. The synthetic view provided by pathways offers a valuable scheme for cancer interpretation in terms of cellular physiology, and comparisons of healthy and corrupted pathways have helped disentangle the disease characteristics generated by a mutation^16^.

As to CRC, it has been found that genes APC, KRAS, SMAD4, TP53 belong to four distinct signaling pathways, respectively WNT, MAPK, TGFβ, and TP53^2^. Each of these pathways corresponds to different functional stages of cell development, comprising stem cell renewal, cell growth and division, control of cell cycle, apoptosis.

In view of their relevance in CRC analysis and treatment, these pathways have been subject to intensive study aimed at reconstructing their topology. To this end, each pathway is often modelled as a graph where the nodes represent the (most important) involved protein species and the edges correspond to mutual relationships described in terms of the chemical interactions of the proteins^2,18,19^. A parallel field of study focuses on pathways dynamics, aiming at determining the time course of the molecular concentrations of the proteins involved. This is achieved by considering the chemical reactions between the elements of the pathway, in order to generate a system of ordinary differential equations (ODEs) for the concentrations of the species involved e.g. in the MAPK pathway^20,21^, the WNT pathway^22^, the SMAD pathways^18^, or the combination of JAK/STAT and MAPK pathways^23^.

Despite being largely employed, pathway analysis presents inherent limitations in that some genes may have multiple functions and thus may be simultaneously involved in several pathways. Specifically, a protein can participate in two or more pathways, thus affecting more cellular functions, which in turn may be influenced by the mutations of genes that belong to different pathways. For example a mutation resulting in the loss of function of a protein may inactivate its related pathway whose function may be restored by the action of molecules belonging to another pathway. These properties may guide the development of targeting drugs^19^. We conclude that interconnections, superpositions, and nonlinearities may put severe limitations on the insight provided by the use of pathways^16^.

To overcome these limitations, pathways may be integrated into chemical reaction networks (CRNs), capable of describing the interactions between many different pathways. This approach usually involves a large number of proteins and many parameters have to be set, such as the rate constants of the pertinent chemical reactions. A detailed description of the topology of a CRN is thus almost unfeasible, and a natural recourse is made to mathematical formulations of the network.

CRNs have been used for example to investigate cancer cell metabolism^24^ and specific issues such as the role of AKT in apoptosis of colorectal carcinoma cells^8,25^. A line of research has been recently developed^26^ whereby cancer is regarded as a robust state resulting from an interaction network whose nodes may represent either single molecules or whole pathways. The general framework has been applied to the mechanistic understanding of various cancer types among which CRC. However, the non-homogenous nature of the nodes of the network makes less accessible the corresponding mathematical formulation.

Focusing on CRC, the most frequent mutations concern proteins belonging to the intracellular signaling network that carries information from the cell membrane to cytosol and nucleus, and backward. A simplified model for such a signaling network has been proposed by Halasz and colleagues^27^ together with a computational approach to estimate the 66 unknown parameters of the model.

Here we consider a more complete CRN containing all the four already mentioned pathways, namely WNT, MAPK, TGFβ, and TP53. The present approach aims at a much more detailed reconstruction of the dynamics of the signal transduction networks, with attention concentrated on the analysis of changes induced by mutations.

Specifically, the starting point of the present analysis is the CRC–CRN originally proposed by Tortolina and colleagues to model the intracellular processing of the information sensed from the environment through the TGFβ, WNT, and EGF families of receptor ligands, at the G1/S transition point of healthy colorectal cells^6,7^. This results in a complex CRN, that comprises 419 proteins involved in 850 chemical reactions forming 10 different interacting pathways. In the original paper^6^, all the parameters of the model, including the values of the rate constants, and the initial values of the protein concentrations, were calibrated on the basis of literature data. We refer to the supplementary material of the aforementioned paper^6^ for a complete description of the procedure used to define the network parameters and for the list of the employed references. Furthermore, the CRC–CRN was validated by means of both simulated and experimental data, studying the response of mutated HCT116 and HT20 CRC lines to perturbing inhibitors.

By applying mass action kinetics and using standard procedures, the CRC–CRN is mapped into a system of ordinary differential equations (ODEs), thus allowing for simulations of the kinetics of the signaling process^8,28,29^. The resulting mathematical model is capable of describing the behavior of healthy physiological networks. In a recent work^30^, we presented a formal mathematical procedure to incorporate within the CRN modeling a healthy cell two particular classes of mutations that result in the loss or in the gain of function of one protein. Here, this general approach is extensively applied to achieve biological insights on the CRC–CRN. To this end, we develop an efficient computational tool for an in silico analysis of the global effects induced on the CRC–CRN by single or multiple simultaneous mutations, chosen among those more significant for CRC progression. Additionally, in this work, we show how the model previously introduced^30^ can be extended to incorporate the targeted action of an inhibitory drug such as Dabrafenib^19^. The biological insights provided by our model are validated by comparing them with results previously published in the literature.

## Results

### The CRC–CRN as a simulation tool for biological analysis of cancer cells

This study was performed focusing our attention to the G1/S transition phase of CRC cells. We have considered a relevant sub-region of the cell's signaling network downstream the constant external growth factors TGFβ, WNT, and EGF, which are all involved in CRC^31^. This has provided the appropriate mathematical framework to simulate the global and quantitative effects of the LoF and GoF mutations most frequently accumulated in CRC cells.

The present approach is inherently global, in that it is capable of considering the combined effects of chemical reactions involving several proteins belonging to the physiological signaling network, as well as of describing the overall changes induced by mutations in a cancer cell. The network provides a comprehensive view of the cascade of reactions, examining them not as components of a single pathway, but in a global and quantitative way. In particular, the mathematical model associated with the CRN provides the equilibrium values of the concentrations of the proteins involved in the network and enables to compare the physiological values with those induced by single or multiple mutations. To this end, the concentrations of the 419 proteins within the CRC–CRN are regarded as the state variables of a dynamical system of 419 ODEs, which describe 850 biochemical reactions following the mass action law and thus depending on as many rate constants. The stable equilibrium states of the network are identified with the stationary states of the system, achieved at large time values; they are computed by numerical integration of the ODEs of the model, after setting the initial values of the protein concentrations.

In principle, simulations through dynamical systems imply dependence of the results on the initial conditions, which in our case consist of a set of 419 parameters describing the values of the protein initial concentrations. As shown in the “Methods” section, the number of the required input parameters actually reduces to 81. Indeed, every equilibrium state is uniquely defined by setting the values of the constant aggregation concentrations within the moiety conservation laws of the network or, equivalently, by fixing a stoichiometric compatibility class.

**Figure 1:**
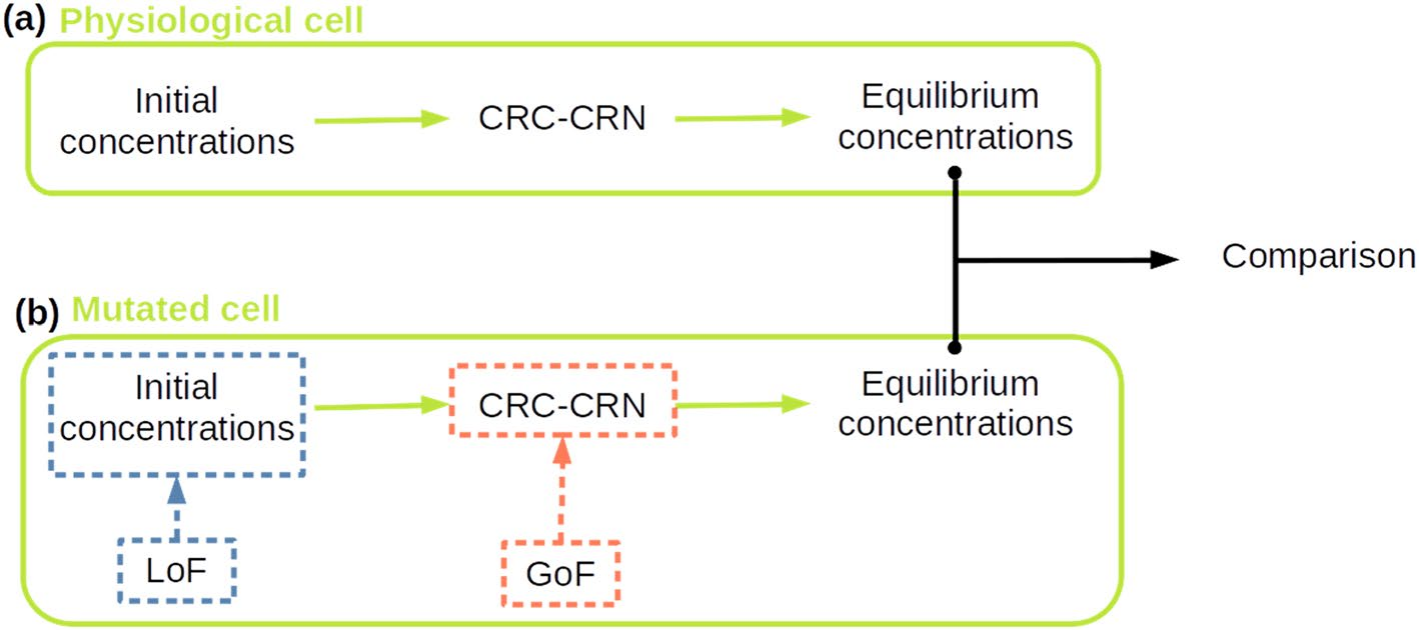
General workflow of the CRC–CRN. (**a**) The values of the protein concentrations at the equilibrium in the physiological cell are computed by integrating the system of ODEs defining our CRC–CRN. (**b**) Any mutation resulting in the loss of function (LoF) of a protein is embedded in our model by modifying the values of the conserved moiety in input. Conversely, mutations causing gain of function (GoF) are implemented by modifying the system of ODEs. The asymptotically stable state of the modified system defines the protein concentrations at the equilibrium in the mutated cell.

Mutations resulting in the LoF or GoF of a selected protein have been incorporated in the CRC–CRN as described in the “Methods” section and synthetically depicted in Fig. 1. By computing the concentrations at the equilibrium of the resulting modified network, we are able to rigorously quantify the impact of the considered mutations on the whole set of proteins involved in the CRC–CRN.

### Simulation of global effects induced by a single-gene mutation

To evaluate whether the CRC–CRN correctly predicts the effects of the mutation of a single gene, we have focused our attention on the mutations that are more common in CRC cancerogenesis^5,9^, namely the GoF of k-Ras, and the LoF of APC, SMAD4, and p53. More specifically, for each one of the four mutations, we have separately computed its impact on the concentrations of the other proteins involved in the CRC network.

**Figure 2:**
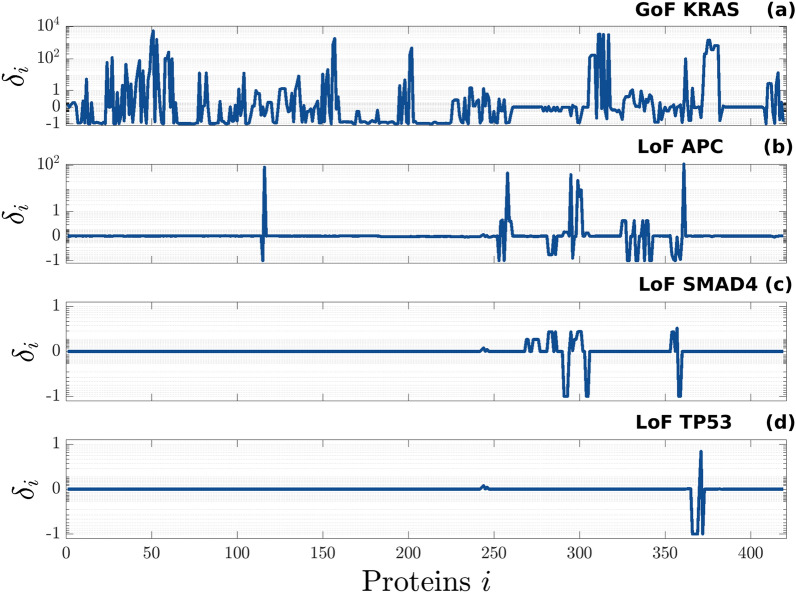
Relative difference between the concentrations at the equilibrium of the mutated and the physiological network. Each panel shows the result obtained by modifying the original CRC–CRN in order to simulate the effect of a different mutation, namely: (**a**) the GoF of KRAS, (**b**) the LoF of APC, (**c**) the LoF of SMAD4, (**d**) the LoF of TP53. For ease of visualization, on the horizontal axis we reported an index *i* = 1, …, 419. In Supplementary Table S1 we report the names of the corresponding proteins *A*_*i*_, while in Supplementary Table S2 we show the list of proteins significantly affected by each one of the four mutations (i.e. |$\delta_{i}$| > 0.03), together with the corresponding value of $\delta_{i}$. For a complete explanation of the abbreviations used for the protein names we refer to the original paper by Tortolina and colleagues^6^.

On the horizontal axis of Fig. 2, we report the proteins $A_{i} ,\;i = 1,{ } \ldots ,{ }419,$ of the network. For each protein, on the vertical axis, we show the relative difference

$$\delta_{i} = \frac{{\tilde{x}_{i}^{e} - x_{i}^{e} }}{{x_{i}^{e} }}$$

where $\tilde{x}_{i}^{e}$ and $x_{i}^{e}$ are the values of the concentration of *A*_*i*_ at the mutated and physiological equilibrium, respectively. The values of $\delta_{i}$ are plotted in bi-symmetric logarithmic scale^32^. A value of $\delta_{i}$ different from zero means that in the mutated network the concentration of the protein $A_{i}$ is either increased ($\delta_{i}$ > 0) or reduced ($\delta_{i}$ < 0). In particular, a value of $\delta_{i}$ equal to − 1 means that the function of protein $A_{i}$ is almost completely stopped in the mutated network. In more general terms, the value of $\delta_{i}$ quantifies the relative change of the protein concentration, normalized by its value in the physiological network, and thus enables identifying which proteins are more sensitive to each one of the four considered mutations.

The results shown in Fig. 2 highlight how mutations may have a different impact on the CRC–CRN when acting on proteins at different levels of the network. Indeed, more than 76% of the proteins are significantly affected by a GoF of k-Ras, a protein upstream in the network, while about only 10.5%, 6.2%, and 1.4% of the proteins are affected by the LoF of APC, SMAD4, and TP53, respectively. Additionally, we found the strongest value of our index $\delta_{i}$. in correspondence with the GoF of k-Ras and the LoF of APC, Fig. 2a,b respectively. In the former case, a few proteins reach a value of $\delta_{i}$ close to $10^{4}$, as a consequence of the fact that they have a very low equilibrium concentration in the physiological network. Consider for example p-p-ERK: in the physiological cell $x_{i}^{e} \simeq 0.017$, while in the network affected by a GoF of k-Ras, $\tilde{x}_{i}^{e} \simeq 91.09$ and thus $\delta_{i} \simeq 5.37 \times 10^{3 }$.

These results suggest that, although KRAS, APC, SMAD4, and TP53 gene mutations are essential events for colorectal cancer development^33^, the downstream effects of their mutation is more evident for the protein upstream in the signaling pathway. Moreover, literature reports that KRAS and APC mutations are the principal causes of the CRC onset, but they are not related to the tumor stage or location^34,35^. Conversely, TP53 mutations seem to increase parallelly with the tumor stage, suggesting that this gene plays a pivotal role in the progression of CRC, more than in the pathology onset^35^. Regarding the role of SMAD4, it displays a pivotal role both in the development and in the progression of CRC^36^. Somatic mutations of SMAD4 are associated with more aggressive tumor biology, poor response to chemotherapy, metastases, and unfavorable overall survival among patients with resectable and unresectable CRC^37,38^. On the other hand, the mutations of upstream proteins of a specific pathway could be counterbalanced by the activation/inhibition of other correlated pathways, while the mutation of downstream proteins, albeit of a minor entity, could determine major damages, since their activity could not be replaced by other pathways.

For each one of the four mutations, Supplementary Table S2 lists the proteins whose concentration significantly changes in the mutated network.

### GoF of PI3K, k-Ras and Raf, and LoF of PTEN and AKT determine an alteration of p53 level

As in the previous subsection, we considered a set of single-gene mutations. The new specific aim is to compare the effects of each mutation on the same target molecule, in order to show how the CRC–CRN can be used to highlight mechanisms that can alter the function of a given protein. In view of its connection with CRC, we focused on p53.

In the previous section, we considered a mutation resulting in the LoF of TP53 and we quantified the alteration induced by such a mutation on the values of the concentrations of all the proteins within our CRC–CRN. As shown in Fig. 2d, since p53 is a downstream protein in our network, the LoF of TP53 alters the concentration of only a few proteins. On the contrary, even when no mutation directly involves the gene TP53, the value of the equilibrium concentration of the protein p53 may be altered by various mutations affecting other proteins located at an upstream level of the CRN^39^. Motivated by these considerations, here we assume that TP53 is not affected by any mutation, and we show how to use the proposed tool to infer the mechanisms altering the concentration of p53 as a consequence of an upstream mutation.

To this end, after selecting a set of mutations to be tested, we computed the equilibrium of the corresponding mutated network and we calculated the relative difference $\delta_{p53}$ between the concentration of p53 in the mutated and the physiological equilibrium. Table 1 shows the value of $\delta_{p53}$ for a set of mutations that significantly impact the concentration of the protein. In detail, the concentration of p53 was reduced by about 0.7 times the value in the physiological network by both the GoF of PI3K and the LoF of PTEN, while it was increased by the GoF of k-Ras and the GoF of Raf. However, the strongest effect is induced by the LoF of AKT in which case the value of $\delta_{p53}$ was found equal to 130.5.

**Table 1:** Relative difference $\delta_{p53}$ of the equilibrium concentrations of p53 induced by a set of single-gene mutations. The first and second rows report the considered mutation and the corresponding value of $\delta_{p53}$, respectively.

Mutation	GoF PI3K	LoF PTEN	GoF KRAS	GoF BRAF	LoF AKT
$\delta_{{p53}}$	− 0.66	− 0.68	1.38	2.28	130.5

**Figure 3:**
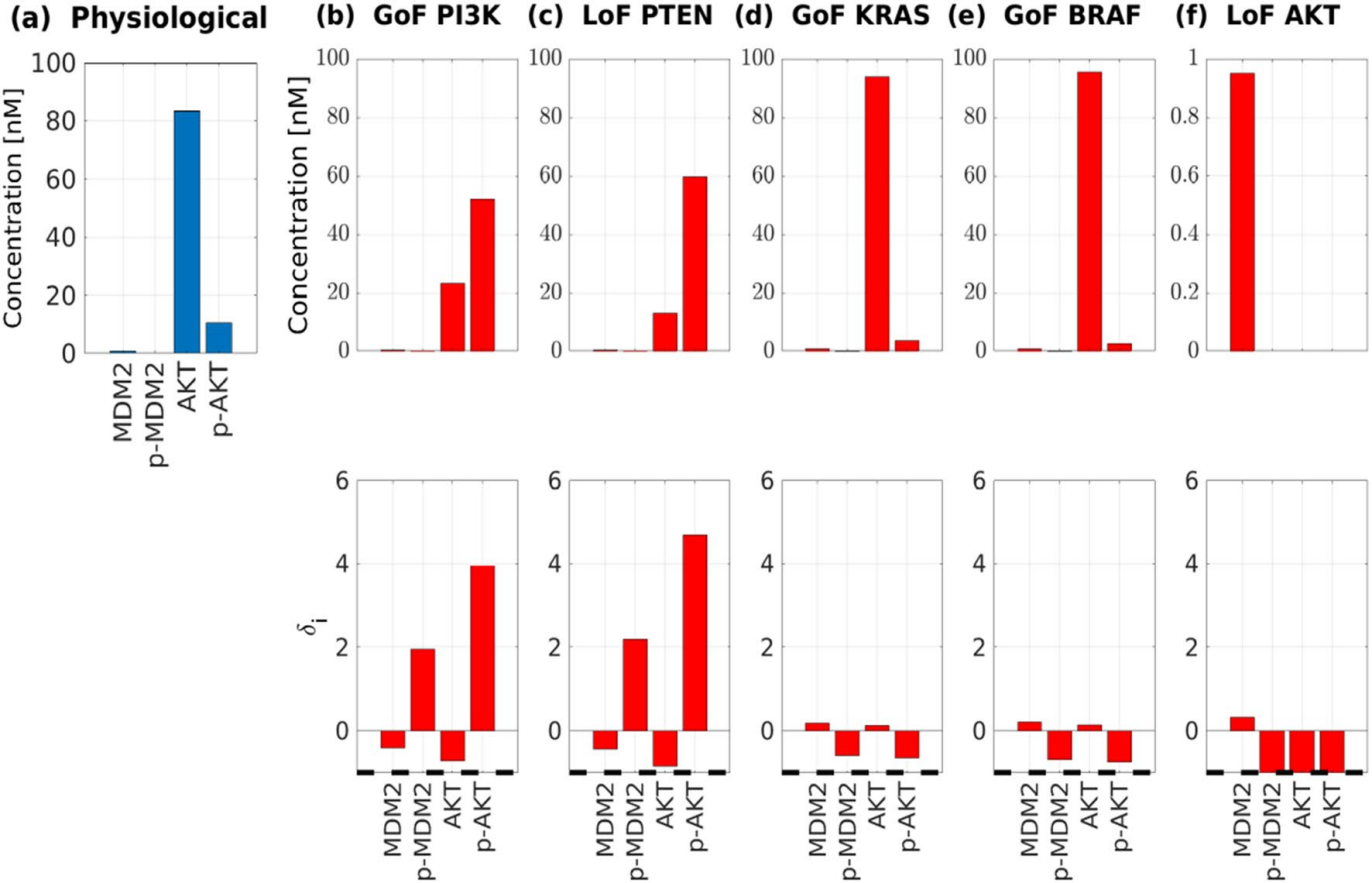
Effects of various single-gene mutations on the value of p53 concentration. (**a**) Value of the concentrations at the equilibrium of the physiological network of the proteins (directly) involved in the degradation of p53, namely MDM2, AKT, and their phosphorylated form p-MDM2 and p-AKT, respectively. (**b**–**f**) Effect on these concentrations of 5 mutations, namely GoF of PI3K, LoF of PTEN, GoF of KRAS, GoF of BRAF, and LoF of AKT. For each mutation in the upper panel, we show the values of the concentrations at the equilibrium of the mutated network. In the lower panel, the corresponding relative difference $\delta_{i}$ is depicted.

To understand the reasons underlying these alterations, we observe that in our CRC–CRN the degradation of p53 is regulated by the phosphorylated form of MDM2, whose activation is in turn regulated by phospho-AKT (p-AKT)^40^. Therefore, in Fig. 3, we show the concentration of MDM2, AKT, and their phosphorylated form p-MDM2 and p-AKT in the physiological network [panel (a)] and the altered values induced by the 5 mutations mentioned in Table 1 [panel (b)–(f)]. We observe that both the GoF of PI3K and the LoF of PTEN promote the phosphorylation of AKT and MDM2 thus speeding the degradation of p53. On the other hand, it was demonstrated on CRC cellular models that activation of the PI3K/AKT pathway inhibits the apoptosis, cell growth, and modulation of cellular metabolism, lowering p53 and PTEN concentration^41,42^. Conversely, the activation of the PTEN pathway decreases the PI3K/AKT-dependent cellular proliferation and regulates the stability of p53^41,43^.

Moreover, our model shows that the GoF of KRAS, and the GoF of BRAF (Fig. 3d,e), downregulate the phosphorylation of AKT, which, in turn, determines the increment of the p53 level. When the LoF of AKT is considered, the phosphorylation of AKT and MDM2 is completely stopped. Indeed, as shown in Fig. 3f in this case the value of $\delta_{i}$ for p-MDM2 and p-AKT is equal to − 1. This explains why in all these mutations the concentration of p53 is increased, but the strongest effect is induced by the LoF of AKT, confirming the relation between RAS/RAF/MEK/ERK signaling axis, the inhibition of AKT pathway, and the intracellular concentration of p53^44^.

None of the considered mutated proteins is related to p53 by a specific, direct chemical reaction. Furthermore, the proteins are rather far from p53 in the network topology and do not belong to the same pathway. Nevertheless, the proposed network approach has disclosed their indirect influence on p53.

### Multiple-gene mutations: effects of pairs of simultaneous mutations on p53 level

Most cancers develop following the accumulation of a series of specific mutations in the cell. Thus, characterizing the impact of the interaction among a group of mutations plays a crucial role in the prediction of tumor progression. Quantifying the combined effect of a set of mutations is not trivial, also when the effects of each single mutation are known, because some mutations may actually induce opposite effects on a given protein. For example, Table 1 shows that the GoF of PI3K and the LoF of PTEN reduce the concentration of p53, while the GoF of k-Ras, the GoF of Raf, and the LoF of AKT individually increase it.

The proposed tool easily allows one to overcome this problem. Indeed, as described in the “Methods” section, the simultaneous action of a group of mutations can be simulated by changing the initial conditions of the system according to each LoF of the group and by removing the reactions associated with each GoF. To show an application, Table 2 synthesizes the results obtained when 6 pairs of mutations are considered, each composed of a mutation that downregulates p53 and a mutation that instead augments its concentration. We observe that when the GoF of PI3K is combined with either the GoF of k-Ras or the GoF of Raf, the concentration of p53 decreases. This may depend on the negative control exerted by the Ras/Raf/MEK/ERK pathway on the downstream AKT signal^44^.

**Table 2:** The relative difference $\delta_{p53}$ of the equilibrium concentrations of p53 induced by pairs of simultaneous mutations. Each element of the table shows the value of $\delta_{p53}$ obtained when the mutations reported in the corresponding row and column are incorporated in the network.

	GoF KRAS	GoF BRAF	LoF AKT
GoF PI3K	− 0.65	− 0.65	130.49
LoF PTEN	− 0.68	− 0.68	130.49

On the contrary, the LoF of AKT prevails on the GoF of PI3K as their combination increases p53 concentration. This could be due to the relationship between PI3K and AKT since PI3K activation is upstream to the AKT signal^45^. Therefore, AKT reduction plays a predominant role on p53 levels regardless of PI3K activation. Analogous results hold for the LoF of PTEN.

Interestingly, the values obtained for the GoF of KRAS and BRAF are the same probably because the proteins codified by these two genes belong to the same pathway, and, in detail, the second is downstream of the first. The same observation can be made also for the GoF of PI3K and the LoF of PTEN.

### Effect of Dabrafenib on the CRC–CRN

Assuming that the CRC–CRN is affected by a mutation resulting in the GoF of k-Ras, we investigated the effect on the mutated network of Dabrafenib, a drug that inhibits Raf activity^19,46^.

To this end, we modelled the drug as a competitive inhibitor^47^, and we added to the CRC–CRN the reversible reaction

$${\text{Raf}} + {\text{Drug}} \mathop{\rightleftarrows} \limits^{{{\text{k}}_{{1{\text{f}}}} }}_{{k_{1r} }} {\text{Raf}}\_{\text{Drug}}$$

where Drug stands for the considered drug, in our example Dabrafenib, and Raf_Drug is the inactive drug-target complex. Additionally, we assume that $k_{1f} \gg k_{1r}$ so that the drug binds almost steadily to the targeted molecules. Specifically, in our simulation we set $k_{1f} = 0.5\;({\text{nM}}\;{\text{s}})^{ - 1}$, which is the average rate constant over all the second-order reactions within the CRC–CRN, and $k_{1r} = 0.005\;{\text{s}}^{ - 1}$. Provided that the ratio between the two rate constants is kept fixed, different values of $k_{1f}$ and $k_{1r}$ will impact the speed at which the modified CRC–CRN reaches the equilibrium but will not significantly alter the final concentration profile.

We then quantified the effect of the drug delivery on the protein concentrations as follows. We modified the CRC–CRN by accounting for both the GoF of k-Ras and the action of the drug targeting Raf. We then integrated the corresponding system of ODEs with initial values of the proteins concentrations equal to the values of the steady-state of the network affected by GoF of k-Ras, and with different values of the drug initial concentration. Specifically, we set

$$x_{{0,{\text{DRUG}}}} = \alpha c$$

where $c = 50$ nM is the total molar concentration at disposal of the proteins within Raf conservation law and $\alpha \in \left\{ {1,\; 0.75, \;0.5,\; 0.25} \right\}$.

**Figure 4:**
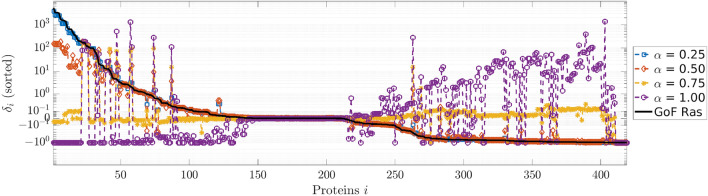
Effects of the drug on the whole protein concentrations profile. The black line represents the relative difference between the concentrations at the equilibrium of the network affected by a GoF of k-Ras and the concentrations at the physiological equilibrium; all the values are sorted in decreasing order. The values $\delta_{i}$ for all the proteins are shown in the second column of Supplementary Table S5. The colored lines represent the relative difference between the equilibrium concentrations obtained after the drug addition in the mutated network and the concentrations in the physiological equilibrium. Each color corresponds to a different value of the initial drug concentration, parameterized by $\alpha$. The values of $\delta_{i}$ when $\alpha = 0.75$ are shown in the third column of Supplementary Table S5.

Figure 4 shows the changes induced by the drug on the whole equilibrium concentration profile. We observe that the drug reaches its highest effectiveness when $\alpha = 0.75$ i.e. when about 37 nM of the drug is delivered to the cell. In this case, the concentrations of most of the proteins involved in the cellular signaling of CRC return to a value close to the steady-state of the physiological condition. Because of the drug action, for most of the proteins, the value of the concentration is restored to a value close to the physiological one. The only exceptions are: (i) Raf, whose function is correctly inhibited; (ii) a group of complexes that involve the activated form of k-Ras, that is still overexpressed; (iii) the complexes that are products of the reactions removed to simulate the GoF of k-Ras, whose function is thus stopped, and a group of corresponding complexes, whose concentration increases so that the total molar concentration within the conservation laws is conserved. We point out that these results hold only when the drug is acting inside the cell. When the drug action is stopped, e.g. by setting to zero the flux rate $k_{1f}$ and by adding a reaction of degradation for the drug to model its consumption, the concentrations of all the species return to the value they have in the mutated cell. The development of a complete pharmacodynamic approach modeling also cell processes such as apoptosis^25,48^ will be the subject of a future study.

Our approach also enabled us to quantify the effect of an under- or over-dosed administration. Indeed, Fig. 4 shows that when $x_{{0,{\text{DRUG}}}}$ is too small, the drug essentially has no impact on the values of the proteins concentrations, while a too high value may result in severe side effects.

**Figure 5:**
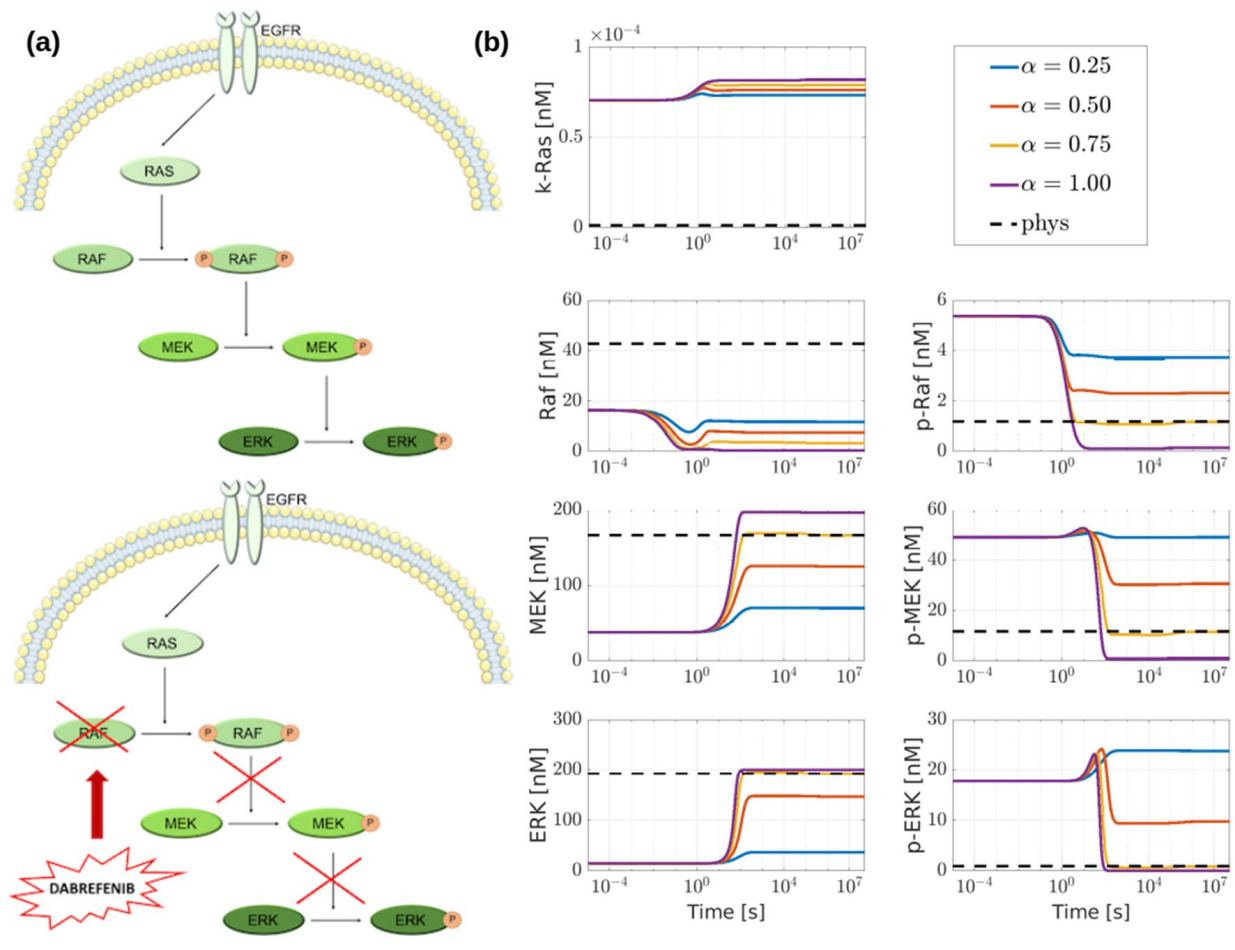
Effects of the drug on the Ras–Raf–MEK–ERK cascade (MAPK pathway). (**a**) Schematic representation of the MAPK pathway extracted from the CRC–CRN and of the changes induced by the drug Dabrafenib modeled as a competitive inhibitor of Raf kinase. (**b**) Time-courses of the concentrations of the proteins within the MAPK cascade obtained by solving the system of ODEs associated to the modified CRC–CRN with the initial state set equal to the equilibrium of the network affected by a GoF mutation of KRAS. Different lines correspond to a different value of the initial concentration of the drug; the black dotted line depicts the value of the protein concentrations in the physiological cell that is taken as reference.

To better understand the mechanism underlying the described network response, we focused on the Ras–Raf–MEK–ERK cascade, i.e. a pathway involving Raf, which is the target of the simulated drug. Acting as a competitive inhibitor, the drug binds Raf to the inactive complex Raf_Drug. As shown in Fig. 5b, this results in a fast decrease of the concentrations of both Raf and its phosphorylated form p-Raf. Interestingly, when $\alpha = 0.75$, p-Raf reaches an equilibrium value equal to that of the physiological network. Shortly thereafter, the reduction of p-Raf concentration downregulates the phosphorylation of MEK and ERK. Indeed, Fig. 5b shows an increase in the concentration of both the proteins at the expense of their phosphorylated form p-MEK and p-ERK. The inhibition of Raf also affects the proteins upstream in the network. For example, as shown in Fig. 5b, the concentration of Ras increases as a result of the drug action, probably as an attempt to induce the activation of Raf. On the other hand, these data based on our model are confirmed by the literature data, which show that the Dabrafenib treatment on CRC cellular model, by inhibiting the Raf activation, increases the Ras level and diminishes the MAPK pathway^49^.

**Figure 6:**
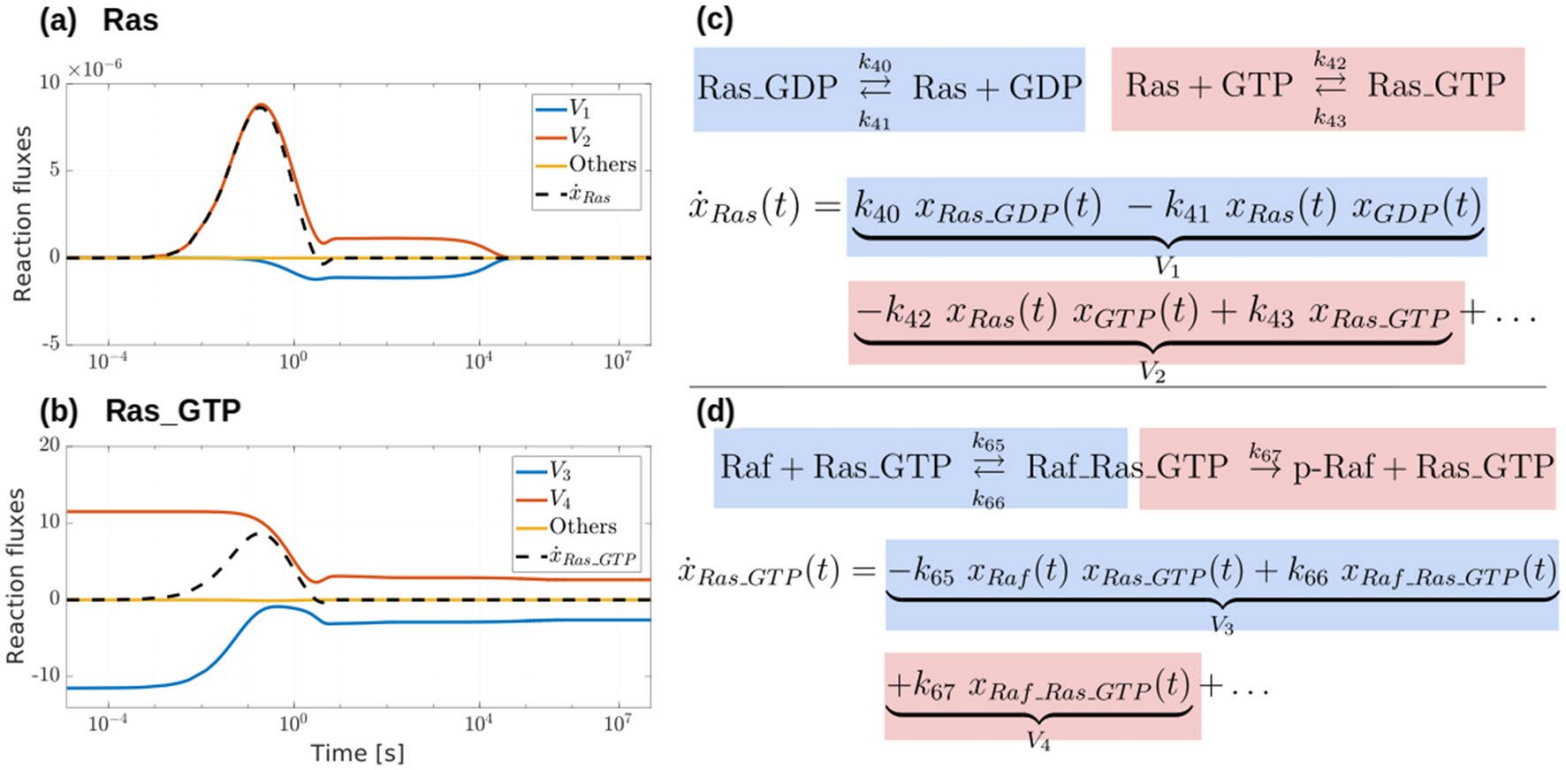
Analysis of the flux rates of the reactions involving Ras and Ras_GTP. (**a**,**b**) Flux rates that contribute to the dynamics of the concentrations of Ras (inactivated form) and Ras_GTP (the activated form) respectively. (**c**,**d**) Mathematical expression of the derivatives of such proteins and corresponding chemical reactions.

To unravel the mechanism underlying this feedback effect, in Fig. 6 we plot the fluxes of the reactions involving Ras and its active form Ras_GTP as functions of time. For the reversible reactions, the sum of the forward and backward fluxes is considered. In Fig. 6c,d we report the fluxes that are significantly different from zero and the corresponding chemical reactions. The figure shows that the network reacts to the reduction of p-Raf concentration by decomposing the complex Raf_Ras_GTP into p-Raf and Ras_GTP. This causes an increase of Ras_GTP concentration that in turns promotes the production of Ras through the decomposition

$${\text{Ras}}\_{\text{GTP}} \to {\text{Ras }} + {\text{ GTP}}{.}$$

As a final remark, we point out that the concentrations of Ras and Raf start changing right after the delivery of the drug and reach an equilibrium value in a rather small time interval. Changes in MEK and ERK concentrations are simultaneous between each other and occur sometime after that k-Ras and Raf reach equilibrium. Since Ras is located upward of Raf in the pathway shown in Fig. 5a, the change in Raf may be regarded as a feedback effect. Changes in MEK and ERK, which occur after Raf has reached equilibrium, may be associated with delay effects.

## Discussion

In this work, we have shown how a computational tool for simulating signal transduction networks can be applied for modeling the information flow inside a CRC cell at the G1/S transition point. To this end, we started from a CRN devised for modeling cell signaling of a healthy colorectal cell^6^ and we exploited a formal mathematical model recently introduced^30^ to quantify the global effects induced on the network by the most common loss and gain of function mutations. Concerning the original approach^30^, in the present work, we extended the model of LoF mutations also to proteins that do not belong to any moiety conservation law but whose synthesis is explicitly included among the chemical reactions of the network. This allowed us to simulate, in particular, a mutation resulting in the LoF of TP53. Additionally, we enlarged the original CRC–CRN to include the drug Dabrafenib, modeled as a competitive inhibitor of Raf. By investigating the effects of Dabrafenib on the Ras–Raf–MEK–ERK cascade, we demonstrated how the analysis of the fluxes of the involved chemical reactions enables disentangling feedback effects within the network.

Results reported in this work show that the proposed model based on a whole molecular interaction map displays several advantages in evaluating the changes in CRC cell signaling induced by mutations and drugs, over classical single-pathway approaches.

First of all, with the proposed mathematical model we are able to quantify the global effects induced on the whole network by local changes due to the mutation of one or more genes. In detail, we considered two particular classes of mutations that result in either the loss or the gain of function of specific proteins of the network. By exploiting this feature, we described the alterations induced on the concentrations of all the proteins within the network by the four mutations more commonly found in CRC cells, namely the LoF of APC, SMAD4, and p53 end the GoF of k-Ras. For each one of the considered mutations, we computed and analyzed the equilibrium states of the physiological and mutated network. In particular, we introduced the relative difference between mutated and physiological equilibrium values of the protein concentrations as a quantitative index to identify which proteins are affected by each mutation, but also to quantify the strength of such effects.

By simultaneously analyzing several interconnected pathways, our tool also allowed us to highlight links between the different proteins that would not be evident by studying a single pathway at a time. Additionally, our framework can be easily adapted to simulate the effect of the occurrence of multiple mutations. As an example, we considered TP53, a gene that has proved to play a pivotal role in CRC progression. In fact, even when TP53 is not directly affected by any mutations, alterations of proteins upstream in the network may induce changes in the concentration of the protein p53. In this work, we quantified the changes induced on the concentration of unmutated p53 by 5 mutations, namely GoF of PI3K, GoF of KRAS, GoF of BRAF, LoF of PTEN, and LoF of AKT, considered one at the time or coupled to simulate the action of two concurrent mutations.

Eventually, we employed our tool to simulate the action of targeted drugs on the CRC cells signaling. In detail, we considered a network affected by a GoF mutation of KRAS, and we analyzed the action of Dabrafenib, modeled as a competitive inhibitor targeting BRAF kinase. By looking at the changes induced on the whole protein concentrations profile, we were able to: (i) obtain a detailed description of the action of the drug on the MAPK pathway, as well as on the other elements of the network; (ii) identify an amount of the drug (37 nM in our simulation) capable of restoring a value of most of the protein concentrations close to that in the physiological network; (iii) propose a reasonable interpretation of the results, in terms of time courses of reaction fluxes.

As extensively discussed in the “Results” section, the results obtained in each scenario have been validated using literature data. For example the different impact that the mutation of KRAS, APC SMAD4 and TP53 have on the whole protein concentration profile is confirmed by the different role that these genes have in CRC onset and/or progression^33–38^. Additionally, our results replicates previous relationships found between TP53 and PI3K pathway and the RAS/RAF/MEK/ERK signaling axis^41–44^. Finally, the results of our flux analysis on the role of Dabrafenib on the MAPK pathway was confirmed by a previous work^49^.

Although preliminary, the results of this work show that the proposed method is capable of predicting the quantitative effects of targeted drugs and thus could represent a valuable support in the design and optimization of novel targeted therapies. In this work, we limited our attention to a kinase inhibitor acting on CRC cells. Future efforts will be devoted to extending the proposed model to different types of drug and cancer cells, and to investigate the interplay between cytoplasmic protein alterations and genomic mutations in order to supply a more comprehensive model of different types of LoF and GoF mutations, including a wider class of mechanisms altering the protein function, such as e.g. copy number variations. Moreover, in this work, we have assumed the parameter of the CRN to be fixed, so that a unique equilibrium point exists once the value of the constant aggregation concentrations within the moiety conservation laws is set. In future work, we will perform a more systematic sensitivity and multistationarity analysis to investigate the robustness of the obtained results to changes in the value of the parameters and, possibly to the addition of reactions modeling the synthesis and degradation of the involved proteins^22,50,51^. Finally, the results of the presented paper have been validated by using literature papers. A more systematic validation through properly designed biological experiments is our next goal.

## Methods

The mathematical formalism used in this work to model the kinetic of the proteins within the CRC–CRN builds on the theoretical results shown in a recent paper^30^ for a general class of CRN. In this section, we summarized such results, highlighting the main methodological improvement introduced in this paper.

### A mathematical model for CRNs

Our CRC–CRN models the G1–S transition in HCT116 and HT29 CRC cell lines as a complex CRN describing the flow of information through 10 interacting pathways^6^. A total of r = 850 reactions involving n = 419 well-mixed proteins were included in our network. The list of all the considered proteins and chemical reactions can be found in Supplementary Table S1 and Supplementary Table S3, respectively. A description of the whole system is also provided in SBML (Systems Biology Markup Language) format^52^ in the Supplementary Data S6.

By applying the law of mass action, the kinetic of the proteins concentrations can be modeled through a system of n ordinary differential equations (ODEs) of the form^29,53,54^

1 $$\dot{\user2{x}} = \user2{S v}({\varvec{x}},\user2{ }\;{\varvec{k}})$$

where ***x = ***$(x_{1} , \ldots , x_{n} )^{T}$ is the state vector identified by the molar concentrations (nM) of the proteins, the superimposed dot denotes the time-derivative, **S** is the constant stoichiometric matrix of size n × r, ${\varvec{v}}({\varvec{x}},\;\user2{ k})$ denotes the time-variant vector of reaction fluxes of length r, and ***k ***$= (k_{1} , \ldots , k_{r} )^{T}$ is the set of known reaction rate constants, whose value is assumed to be fixed in this work and can be found in Supplementary Table S3. In Eq. (1) we have assumed that all the molecular exchanges between the cell and the environment are encoded in the stoichiometric matrix.

In this work, we are mainly interested in characterizing the state of the system when the network reaches equilibrium. To this end, after setting the initial values of the protein concentrations, we integrate the system of ODEs (1), using the Matlab tool ode15s^55^, and we take the asymptotic value of the obtained solution as the equilibrium point.

Our model of LoF and GoF mutations builds on the analysis of the moiety conservation laws of the system. Each conservation law identifies a group of proteins whose aggregate concentrations do not change with time and is formally defined as a set of positive, integer coefficients **γ** = (γ_1_, …, γ_n_)^T^ such that the product **γ**^T^ ***x***(*t*) remains fixed over the simulated concentrations dynamic.

A set of generators for all the moiety conservation laws of the system can be computed by studying the left null space of the stoichiometric matrix^56,57^. By applying this procedure to the CRC–CRN we obtained 81 independent moiety conservation laws involving all the proteins within the network but 10. The latter are either proteins that undergo degradation or proteins that have direct contact with the external environment. We observe that in the CRC–CRN we modelled the direct degradation and synthesis of only a limited number of the involved proteins. Specifically, the proteins to be degraded/synthetized are chosen based on biological motivations (such as p53 degradation) or mathematical requirements to guarantee that all the proteins reach an unique equilibrium state. This approximation is often found in the literature^27,58^, however, future effort will be devoted to enlarge the CRC–CRN so as to account for the fact that proteins are being continuously degraded and synthesized. In particular a thorough analysis to detect possible multistability of the enlarged network will be performed^51,59,60^.

The importance of conservation laws is twofold. On the one hand, we have numerically verified^30^ that, once the values of the reaction rate constant ***k*** have been fixed, the system of ODEs (1) admits a unique equilibrium on the set

$$SC({\mathbf{c}}) = \{ {\mathbf{x}} \in R^{n}\; \text{s.t.}\; \boldsymbol{\gamma}_{j}^{T} {\mathbf{x}} = c_{j} ,\;\; j = 1, \, \ldots , \, p\}$$

where **γ**_1_, …, **γ**_p_ are the p = 81 independent constant generators of the moiety conservation laws and ***c ***$= (c_{1} , \ldots , c_{p} )$ is the vector of the corresponding constant aggregation concentrations. The set *SC*(***c***) is called the stoichiometric compatibility class. As we shall see in the next section, different stoichiometric compatibility classes define different biological states of the network (healthy or mutated). On the other hand, we made use of the conservation laws to simplify the input parameters required by our simulation tool for computing the equilibrium states. In fact, the equilibrium state of the network, mimicking either a physiological or a mutated cell, is fully characterized by assigning the set ***c*** of the constant aggregate concentrations. Indeed, the value of ***c ***defines a unique stoichiometric compatibility class and all initial states belonging to the same class lead to the same stationary state. Therefore, every equilibrium state corresponds to a set of 81 constants, and conversely^30^.

### LoF mutations

By exploiting the mathematical framework developed in our previous work^30^, we simulated the effect of a LoF of APC, AKT, SMAD4, and PTEN. Since each one of these proteins is involved in only one conservation law, their LoF mutations are implemented by setting to zero the total concentration at disposal of the corresponding conservation law. In practice, this is achieved by projecting the initial concentration values describing the physiological cell into a new initial state where the concentrations of the mutated protein and of all its compounds are set to zero.

In this work, we also simulated the LoF of p53 by downregulating its production. Since p53 undergoes degradation, it is not involved in any concentration law and thus the previous framework does not hold. However, in our CRC–CRN the production of p53 is modeled by the presence of an auxiliary variable, called *p53_generator*, whose concentration is assumed to be constant to model the presence of a pool that constantly feeds the production of p53. In the mutated cell, such production is stopped by setting to zero the concentration of *p53_generator*. The production of p53 can be equivalently modelled by a pseudo reaction from the null complex to p53^53^. In this case, the LoF of p53 could be implemented by setting to zero the corresponding rate constant.

All the LoF mutations considered in this work simulate the effect of null mutations where the function of the mutated proteins is totally lost and the concentrations of the related molecules vanish. However, mutations with a different degree of loss of function can be easily achieved by setting the amount of available total concentration to a value lower than the one in the physiological cell, but different from zero.

### GoF mutations

In this work, we quantified the effect of mutations resulting in the GoF of k-Ras, Raf, PI3K, and Betacatenin. The GoF of a given protein is induced by removing from the CRC–CRN all the reactions involved in its deactivation^30^.

As an example, the dephosphorylation of Raf is modeled in our CRC–CRN through the set of reactions

$${\text{p-Raf }} + {\text{ Pase1}} \mathop{\rightleftarrows }\limits_{{k_{1r} }}^{{\text{k}}_{1{\text{f}}} } {\text{p-Raf}}\_{\text{Pase1}}\mathop{\rightarrow}\limits_{} ^{{k_{2} }} {\text{Raf }} + {\text{ Pase1}}$$

where p-Raf is the activated form of Raf, consisting in the phosphorylation of a specific amino acid.

A GoF of Raf is induced by setting to zero the rate constants in the two forward reactions, namely, we set $k_{1f} = k_{2} = 0$. This results in a novel set of n ODEs

2 $$\dot{\user2{x}} = \widetilde{{{\varvec{S}} }}\user2{ v}({\varvec{x}},\user2{ }\;{\varvec{k}})$$

where the stoichiometric matrix $\widetilde{{{\varvec{S}} }}$ is defined by setting to zero the two columns of the matrix ***S*** in (1) corresponding to the deleted chemical reactions. By removing only the two forward reactions, the deactivation of p-Raf is completely blocked while the rank of the novel stoichiometric matrix is kept equal to that of the original stoichiometric matrix. As a consequence, the reduced system of ODEs described by Eq. (2) maintains the same conservation laws of the original system (1).

Supplementary Table S4 shows the list of reactions removed when implementing each one of the GoF mutations considered in this paper, namely the GoF of BRAF, the GoF of k-Ras, and the GoF of PI3K.

Similarly to what we did for the LoF mutations, mutations resulting in different degrees of gain of function of the considered protein, can be modeled by reducing the value of the rate constants $k_{1f }$ and $k_{2}$. By doing so, the deactivation of Raf still takes place, but at a slower speed than in the physiological cell.

### Combination of multiple LoF and GoF mutations

Consider a cell affected by a number of mutations each one of them resulting in the loss or gain of function of a specific protein. As we have shown in a previous work^30^, our framework allows us quantifying the simultaneous effect of all mutations.

Specifically, each LoF mutation entails a change in the value of the total concentrations provided to the algorithm. Instead, to account for the GoF mutations we modify system (1) by reducing or zeroing the value of the rate constants corresponding to reactions involved in the deactivation of the proteins affected by this type of mutations. The value of the protein concentrations at the equilibrium is computed by integrating the modified system of ODEs with initial conditions defined according to the constraints imposed by the LoF mutations.

Importantly, the same set of steady-state values would have been obtained by starting from the system modeling the physiological state of the cell and iteratively computing the equilibrium of the modified system accounting for an increasing number of mutations, regardless of their order.

## Supplementary Information

Supplementary Information 1.

Supplementary Information 2.

Supplementary Information 3.

Supplementary Information 4.

Supplementary Information 5.

Supplementary Information 6.

Supplementary Information 7.

## Data Availability

All relevant data and codes for reproducing this study are deposited on GitHub at https://github.com/theMIDAgroup/CRC_CRN.git.
