# Supplementary material for: Computational quantification of global effects induced by mutations and drugs in signaling networks of colorectal cancer cells
Source: Sci Rep. 2021 Oct 1;11:19602. doi: 10.1038/s41598-021-99073-7 (PMC8486743; doi:10.1038/s41598-021-99073-7)
Supplement: Supplementary file 7 — Supplementary Information 7. [file 41598_2021_99073_MOESM7_ESM.pdf]

Supplementary information for

Computational quantification of global effects induced by mutations and drugs in signaling networks of colorectal cancer cells.

Sara Sommariva[1*], Giacomo Caviglia[1] , Silvia Ravera[2] , Francesco Frassoni[1] , Federico Benvenuto[1] , Lorenzo Tortolina[3] , Nicoletta Castagnino[3] , Silvio Parodi[3] , and Michele Piana[1]

[1]Dipartimento di Matematica, Università di Genova, via Dodecaneso 35, 16146 Genova, Italy
[2]Dipartimento di Medicina Sperimentale, Università di Genova, Via De Toni 14, 16132 Genova, Italy
[3]Dipartimento di Medicina Interna, Università di Genova, via Leon Battista Alberti 2, 16132 Genova, Italy

*sommariva@dima.unige.it

Supplementary information includes 5 tables described below.

Supplementary Table S1. Table of the proteins A_i, $i = 1, \ldots, 419$, involved in the CRC-CRN related to Fig. 2. In the third column we reported the values of the initial concentrations used for computing the equilibrium state of the healthy network.

Supplementary Table S2. Table of the proteins significantly affected (i.e. $|\delta_i| > 0.03$) by the mutations related to Fig. 2.

Supplementary Table S3. Table of the chemical reactions and of the values of the rate constants involved in the CRC-CRN related to the Methods section, paragraph 'A mathematical model for CRNs'.

Supplementary Table S4. Table of the chemical reactions removed when implemented each of the GoF mutations related to Methods section, paragraph GoF mutations.

Supplementary Table S5. Table related to Fig. 4, and listing (i) the relative difference between the concentrations at the equilibrium of the network affected by a GoF of k-Ras and the concentrations at the physiological equilibrium and (ii) the relative difference between the equilibrium concentrations obtained after that about 37 nM of drug has been incorporated in the mutated network and the concentrations in the physiological equilibrium.

Supplementary Data S6. Whole chemical reaction network for colorectal cancer (CRC-CRN) in Systems Biology Markup Language (SBML) format.
